# Supplementary material for: Differences in the long-term course of post-COVID-19 symptoms in adults and children across epidemic periods: A retrospective cohort study in Japan, 2020–2024
Source: PLoS One. 2026 May 8;21(5):e0348954. doi: 10.1371/journal.pone.0348954 (PMC13155679; doi:10.1371/journal.pone.0348954)
Supplement: S1 Fig — (PDF) [file pone.0348954.s003.pdf]

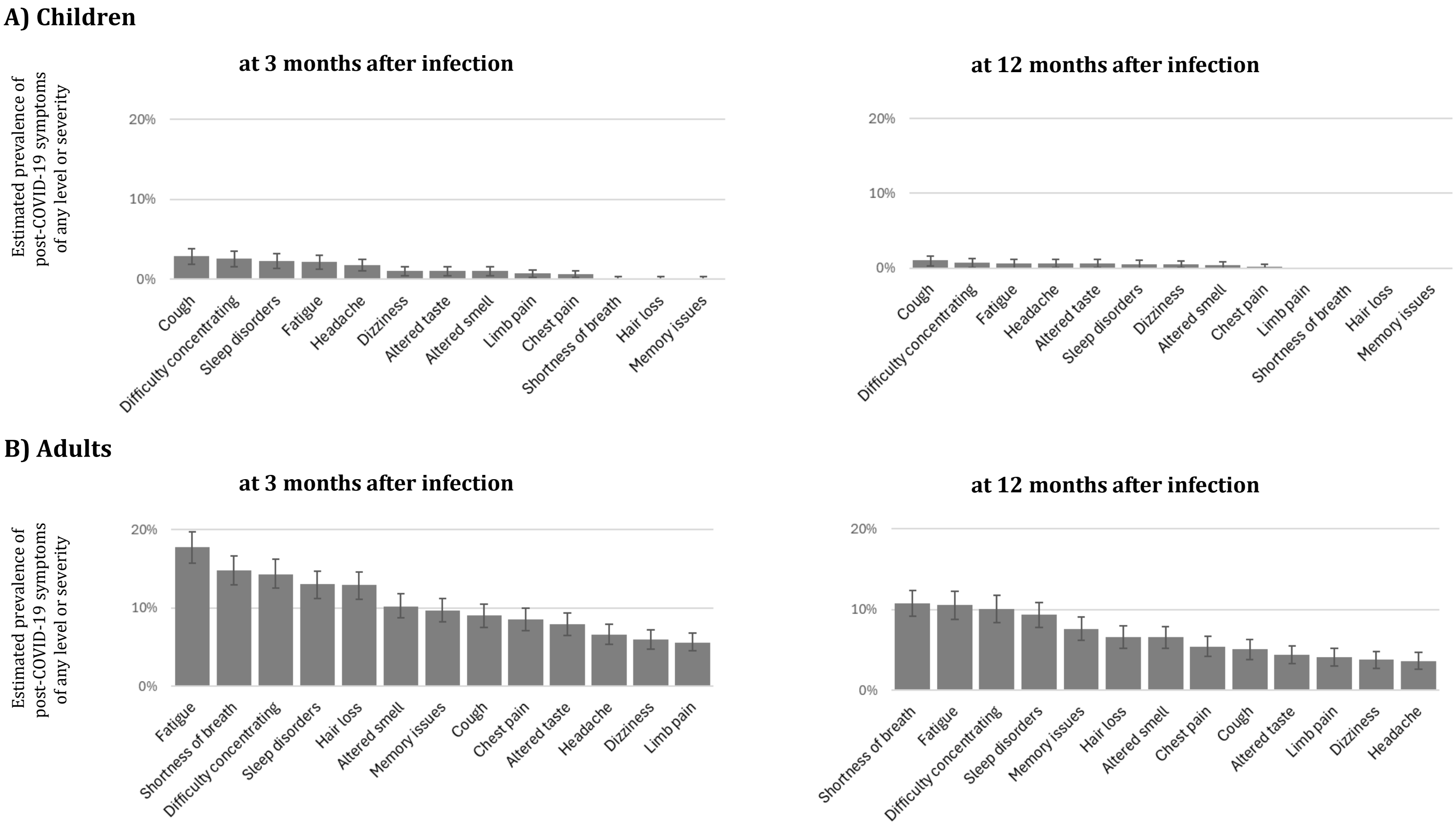

S1 Fig. Estimated prevalence of 13 post-COVID-19 symptoms at 3 and 12 months after infection, by age group

Prevalence was estimated at two common time points (3 and 12 months after infection) using the Turnbull method with step-function alignment of survival curves.
Bars represent prevalence estimates with 95% confidence intervals. Symptoms on the x-axis are ordered by prevalence within each age group.
Left panels: prevalence estimates at 3 months after acute infection; right panels: prevalence estimates at 12 months after acute infection.
A total of 1,165 children and 1,524 adults were included.
