# Supplementary material for: Differences in the long-term course of post-COVID-19 symptoms in adults and children across epidemic periods: A retrospective cohort study in Japan, 2020–2024
Source: PLoS One. 2026 May 8;21(5):e0348954. doi: 10.1371/journal.pone.0348954 (PMC13155679; doi:10.1371/journal.pone.0348954)
Supplement: Attachment — Submitted filename: Response to Reviewers_260131.pdf [file pone.0348954.s005.pdf]

Response to Reviewers

We sincerely thank the Academic Editor and the reviewers for their thorough evaluation of our manuscript and for the detailed and constructive comments provided. We appreciate their careful assessment of our study and their valuable suggestions, which have helped us to improve the clarity, rigor, and interpretation of the manuscript.

We have carefully considered all comments and have revised the manuscript accordingly. Below, we provide a point-by-point response to each comment. All changes made to the manuscript are indicated in the revised version with tracked changes.

Reviewer #1

This study with the title "Differences in the Long-term Course of Post-COVID-19 Symptoms in Adults and Children across Epidemic Periods: A Retrospective Cohort Study in Japan, 2020–2024" gives an insight about the long-term effects of Covid 19 and its co-relation with patient age. But the study seems to have a few unanswered questions. The authors need to discuss some of these questions: -

1. *The authors discussed the different strains of SARS Covid-19 and their long-term symptoms in different patient groups. Do these patients get more than one strain or are they affected by multiple waves of the infection at different time points?*

Response:

We thank the reviewer for this important and thoughtful comment. In this study, the analytic unit was the infection episode recorded at the participating hospital, which was treated as the index infection for the purposes of our analyses.

During the early phase of the COVID-19 pandemic in Japan, medical care for confirmed cases, including both outpatient visits and hospitalizations, was administratively coordinated by local governments. In Hiroshima, patients were largely concentrated at the participating hospital during this period. Therefore, for infections occurring in the early epidemic phases, it is reasonably likely that the recorded cases represent first infections.

In contrast, from 2022 onward, particularly during the Omicron-dominant periods, the rapid expansion of infections resulted in patients seeking care at a variety of medical institutions. Accordingly, we acknowledge the possibility that some individuals may have experienced a prior infection treated at another facility and subsequently visited the participating hospital for a later infection episode.

For participants included in the follow-up survey of the original cohort, we explicitly instructed respondents to report the duration of symptoms persisting from the previously surveyed infection episode and not to include symptoms newly developed after subsequent infections.

For the additional cohort of individuals infected during the Omicron-dominant period in 2024, the survey questions were structured to ask specifically about the timing of infection and symptoms associated with that infection episode. Thus, even if an individual had experienced a previous infection, the reported symptoms are expected to primarily reflect those associated with the Omicron 2024 infection.

Based on the reviewer’s suggestion, we have revised the Methods and Discussion sections as described below.

(Method) Page7, Line113-115

Among participants in the previous survey (November 2022–March 2023), 466 individuals (403 adults and 63 children) who still reported post-COVID-19 symptoms were followed up to collect additional information. **For participants in the follow-up survey of the original**

cohort, respondents were explicitly instructed to report only symptoms persisting from the previously surveyed infection episode and not to include symptoms newly developed after subsequent infections. The current survey was conducted between December 2024 and March 2025, and the data obtained were integrated into the database established in the previous study for analysis.

(Discussion, Limitations) Page20, Line351-356
Sixth, although the analysis was based on infection episodes recorded at the participating hospital, information on prior infections treated at other medical institutions was not fully available, particularly during later epidemic periods. Therefore, some participants may have experienced reinfections before the index episode analyzed in this study. While the survey design aimed to minimize misclassification by focusing on symptoms attributable to the specified infection episode, residual uncertainty regarding reinfections cannot be completely excluded.

2. *The study is based on a survey that includes children who can have different experiences than the adult involved. How did the authors provide more information and support to the children for specific responses.*

Response:
We thank the reviewer for this important comment. In this study, responses for pediatric participants were obtained primarily through proxy responses provided by parents or legal guardians, which is a standard approach in pediatric survey research.
Parents or guardians were asked to complete the questionnaire based on their observations of the child's symptoms and daily functioning. When children were able to communicate their symptoms, parents or guardians were allowed to consult with them while completing the survey. This approach was adopted to ensure accurate reporting while taking into account the child's age, developmental stage, and ability to respond independently.
We have clarified this point in the Methods section of the revised manuscript.

(Method) Page7, Line125-128
For pediatric participants, questionnaire responses were obtained primarily through proxy responses provided by parents or legal guardians. Parents or guardians completed the survey based on their observations, with input from the child when appropriate.

3. *In figure 2, it is interesting to find old versions of viruses showing more severity in symptoms than the later mutants arise. Is it consistent with time and people in each survey.*

Response:
We thank the reviewer for this insightful comment. Figure 2 presents interval-censored survival curves generated using a consistent analytic framework (Turnbull method) across all epidemic periods, stratified by age group (adults and children). These curves are descriptive in nature and therefore reflect the observed distributions of symptom resolution, incorporating differences in participant characteristics, disease severity, and sample size across epidemic periods.
To account for these differences, we conducted adjusted analyses using Cox proportional hazards models. Importantly, even after adjustment for relevant covariates, the association between epidemic period and symptom resolution remained statistically significant.
We have clarified the descriptive role of Figure 2 and the complementary role of the adjusted Cox

models in the Discussion section of the revised manuscript to avoid overinterpretation of variant-specific effects.

(Discussion) Page17, Line289-295

Before interpreting these differences, it should be noted that the comparisons across epidemic periods in this study represent period-based associations rather than direct causal effects of specific viral variants. Although the interval-censored survival curves were generated using a uniform analytic framework across all periods, they reflect underlying differences in participant composition, clinical severity, and sample size across epidemic periods. Importantly, however, the association between epidemic period and symptom resolution remained statistically significant after adjustment for relevant covariates in the Cox proportional hazards models.

4. *I did not find any information about the co-infection or any other illness that happened to the responders during the survey duration.*

Response:

We thank the reviewer for raising this important point. Information on co-infections or other intercurrent illnesses during the survey period was not systematically collected in this study.
However, the questionnaire was designed to specifically ask respondents to report symptoms that they perceived as having been caused by their COVID-19 infection and that persisted thereafter. Participants were instructed to focus on symptoms attributable to COVID-19 rather than newly developed symptoms due to other illnesses.
We acknowledge that self-reported attribution of symptoms may not be perfect and that some degree of misclassification cannot be entirely excluded. This limitation has been clarified in the Discussion section of the revised manuscript.

(Discussion, Limitations) Page20, Line357-361

In addition, information on co-infections or other intercurrent illnesses during the survey period was not systematically collected. Although the questionnaire explicitly asked respondents to report symptoms perceived as being caused by COVID-19 and persisting thereafter, attribution of symptoms based on self-report may not be perfect, and some degree of misclassification related to non–COVID-19 conditions cannot be completely excluded.

Reviewer #2

This study analyzes post-COVID-19 symptoms (PCC) in Japan across variants from Wild-type to Omicron in 2024. Including both adults and children fills a research gap. Using interval-censored survival analysis (Turnbull method) enhances confidence in duration estimates.

The authors note that vaccination history was not significantly associated with symptoms lasting more than three months in their earlier cohort analysis. However, they acknowledge that external studies (e.g., among U.S. veterans) observed a notable risk reduction with vaccination. Additional discussion of why this cohort might differ, such as the timing of vaccination relative to infection periods, especially during high-risk periods like the Delta variant, would be valuable.

Response:

We thank the reviewer for this important and constructive suggestion. In our earlier cohort analysis, vaccination history was not significantly associated with symptoms persisting beyond three months. One possible explanation, as discussed in that prior study, is that our study population included relatively few severe acute COVID-19 cases, which may have attenuated the observable protective effect of vaccination on long-term symptoms.

We have added a brief discussion of these points to the Discussion section of the revised manuscript.

(Discussion, Limitations) Page19-20, Line344-351

Fifth, the effects of vaccination were not directly evaluated in the present study. In our previous analysis using the same cohort(12), vaccination history was not significantly associated with post-COVID-19 symptoms persisting for more than three months after infection in multivariable models. **One possible explanation is that the study population included relatively few severe acute COVID-19 cases, which may have limited the ability to detect a protective effect of vaccination on long-term outcomes.** Although other studies have reported an association between vaccination and a reduced risk of long COVID in different settings (5), such an association was not observed in our cohort.

The manuscript notes that although some children (1.9% to 4.1% depending on the period) experienced symptoms beyond two years, these did not disrupt daily life. Clarifying which symptoms, such as cough or difficulty concentrating, were most persistent would help create a clearer clinical picture.

Response:

We thank the reviewer for this helpful and insightful comment. As suggested, we have clarified the clinical profile of symptoms persisting beyond two years among children. Specifically, we have added a brief description to the Results section indicating which symptoms remained in these cases, noting that they included fatigue, headache, dizziness, cough, difficulty concentrating, and sleep disorders, and that these symptoms did not interfere with daily activities.

(Results) Page13, Line232-233

Symptoms that persisted for more than 2 years showed little further resolution thereafter in both adults and children; however, in children, these symptoms did not interfere with daily activities **and included fatigue, headache, dizziness, cough, difficulty concentrating, and sleep disorders.**

The authors correctly point out that participants with ongoing symptoms might be more likely to respond.

While they argue this does not skew comparisons across periods, they should explicitly note that the overall prevalence such as 47% at six months for Delta might represent an upper limit due to this potential bias.

Response:
We thank the reviewer for this important and helpful comment. As noted, individuals with persistent symptoms may have been more likely to respond to the survey, which could lead to an overestimation of the absolute prevalence of post-COVID-19 symptoms. While we believe that this response bias is unlikely to substantially affect comparisons across epidemic periods, we agree that the overall prevalence estimates, such as the 47% prevalence at six months during the Delta period, should be interpreted as potential upper limits rather than precise population-level estimates.

(Discussion, Limitations) Page19, Line326-328
First, selection bias may have occurred, as participants with persistent symptoms may have been more likely to respond to the survey, potentially leading to an overestimation of overall prevalence. However, because data collection and analysis were conducted in a uniform manner across all groups, this bias is unlikely to have substantially affected comparisons between epidemic periods. **Therefore, the absolute prevalence estimates reported in this study, including the 47% prevalence at six months during the Delta period, should be interpreted as potential upper limits rather than precise population-level estimates.**

We have clarified this point explicitly in the Discussion section of the revised manuscript.
The results indicate that newer sublineages (JN.1, etc.) are not significantly different from early Omicron variants (BA.1/2/5) in symptom duration. This is a novel finding and could be more prominently highlighted in the Conclusion to underscore the current relevance of the study.

Response:
We thank the reviewer for this insightful and encouraging comment. We agree that the finding that symptom duration associated with newer Omicron sublineages, including JN.1, did not substantially differ from that observed during the early Omicron period represents an important and timely contribution.
In response to this suggestion, we have revised the Conclusion section to more prominently highlight this finding and to emphasize the current relevance of our study.

(Discussion, Conclusion) Page21, Line375-378
Importantly, our findings indicate that the frequency and duration of post-COVID-19 symptoms associated with recent Omicron sublineages circulating in 2024, including JN.1, were not substantially different from those observed during the earlier Omicron period, underscoring the current clinical relevance of this study.

Reviewer #3

This manuscript represents a strong and policy-relevant contribution to the long-COVID literature, particularly regarding variant-specific and age-specific trajectories. The suggested revisions are clarificatory rather than fundamental and can be addressed without additional analyses.

1. *Figure 1: Flow diagram of the study population. Among all individuals diagnosed with COVID-19 at the participating hospital between March 2020 and July 2022 (N = 6,551), adults (n = 3,748) and children (n = 2,830) ; Adults and children number together isn't added up to 6551. Also, the final analysis population comprised 2,689 participants, including 1,524 adults and 1,165 children. These totals represent the combined analytic cohort derived from respondents to the initial survey (November 2022–March 2023) and newly diagnosed cases enrolled in 2024, However, the adult and pediatric totals do not correspond to a simple arithmetic sum of previously reported and newly recruited cases.*

    Response:
    We thank the reviewer for carefully identifying these inconsistencies in Figure 1.
    First, we acknowledge that the numbers in the flow diagram were incorrectly reported in the original version. Specifically, among all individuals diagnosed with COVID-19 at the participating hospital between March 2020 and July 2022 (N = 6,551), the correct breakdown is adults (n = 3,748) and children (n = 2,803), rather than 2,830. This error has been corrected in the revised Figure 1.
    Second, regarding the composition of participants who responded to the initial survey conducted between November 2022 and March 2023, the correct numbers are 2,421 respondents, comprising 1,387 adults and 1,034 children. These values have also been corrected in Figure 1.
    We note that these figures differ slightly from those reported in our previous publication using the same cohort, where age group classification was based on age at the time of questionnaire response.
    We appreciate the reviewer's careful review and helpful comments, which have improved the accuracy and clarity of the figure.

2. *Explicitly clarify why vaccination was not included? It might really have an effect.*

    Response:
    We thank the reviewer for this important comment. As noted, vaccination may have an effect on the risk and duration of post-COVID-19 symptoms. However, vaccination status was not directly evaluated in the present study.
    As clarified in the revised manuscript (Discussion, Limitations), our previous analysis using the same cohort did not find a significant association between vaccination history and post-COVID-19 symptoms persisting beyond three months in multivariable models. One possible explanation, as discussed, is that the study population included relatively few severe acute COVID-19 cases, which may have limited the ability to detect a protective effect of vaccination on long-term outcomes.
    We have explicitly acknowledged this point as a limitation in the Discussion section and noted that evidence from other settings has suggested a protective association between vaccination and long COVID.

(Discussion, Limitations) Page19-20, Line341-351

Fifth, the effects of vaccination were not directly evaluated in the present study. In our previous analysis using the same cohort(12), vaccination history was not significantly associated with post-COVID-19 symptoms persisting for more than three months after infection in multivariable models. **One possible explanation is that the study population included relatively few severe acute COVID-19 cases, which may have limited the ability to detect a protective effect of vaccination on long-term outcomes. Although other studies have reported an association between vaccination and a reduced risk of long COVID in different settings (5), such an association was not observed in our cohort.**

3. *Clarify how "did not interfere with daily activities" was operationalized in children (parent-reported? school attendance?).*

Response:
We thank the reviewer for this helpful comment. In this study, interference with daily activities was assessed using a question asking whether each symptom interfered with daily life. The same wording was used for adults and children.
For pediatric participants, responses were obtained primarily through proxy reporting by parents or legal guardians, based on their overall judgment of whether the symptom interfered with the child's daily life. We have clarified this point in the Methods section of the revised manuscript.

(Method) Page7, Line125-128
For each symptom, participants were also asked whether it interfered with daily life. Because precise recall of symptom duration was difficult, responses were collected in ranges of several months. **For pediatric participants, questionnaire responses were obtained primarily through proxy responses provided by parents or legal guardians. Parents or guardians completed the survey based on their observations, with input from the child when appropriate.**

4. *The conclusion could include one sentence emphasizing relevance to other high-income settings with similar healthcare access, while acknowledging cultural/system differences.*

Response:
We thank the reviewer for this helpful suggestion. In response, we have added one sentence to the Conclusion to note that our findings may be relevant to other high-income settings with similar access to healthcare, while acknowledging that differences in healthcare systems and cultural contexts may limit generalizability.

(Discussion, Conclusion) Page21, Line379-381
These findings may also be relevant to other high-income settings with broadly accessible healthcare systems, although differences in healthcare structures and cultural contexts should be considered when interpreting the results.

Reviewer #4

This is a well-conducted retrospective cohort study addressing an important and timely question regarding the long-term course of post-COVID-19 symptoms across epidemic periods and age groups. The long follow-up (extending beyond two years), inclusion of both adults and children within the same framework, and use of interval-censored survival analysis are clear strengths.

Major Comments:

1. *Selection Bias: The study population consists of survey respondents, with response rates around 35–50% across waves.*

   Response:

   We thank the reviewer for this important comment regarding potential selection bias. We acknowledge that the study population consisted of survey respondents, with response rates of approximately 35–50% across survey waves, and that individuals with persistent symptoms may have been more likely to participate.

   In response to this concern, we have revised the Discussion section of the manuscript to explicitly address the potential impact of selection bias. As described in the revised Discussion (Limitations), this response pattern may lead to an overestimation of the absolute prevalence of post-COVID-19 symptoms. However, because data collection procedures and analytic methods were applied uniformly across epidemic periods and age groups, we believe that this bias is unlikely to have substantially affected comparisons across epidemic periods.

   We further clarified in the Discussion that the reported prevalence estimates should be interpreted as potential upper limits rather than precise population-level estimates.

   (Discussion, Limitations) Page19, Line326-328

   First, selection bias may have occurred, as participants with persistent symptoms may have been more likely to respond to the survey, potentially leading to an overestimation of overall prevalence. However, because data collection and analysis were conducted in a uniform manner across all groups, this bias is unlikely to have substantially affected comparisons between epidemic periods. **Therefore, the absolute prevalence estimates reported in this study, including the 47% prevalence at six months during the Delta period, should be interpreted as potential upper limits rather than precise population-level estimates.**

2. *Persistence Beyond Two Years: The manuscript states that symptoms persisting beyond two years showed "little further resolution."*

   Response:

   We thank the reviewer for this important comment. The statement regarding limited resolution beyond two years was intended to describe the observed pattern within the available follow-up period. In response to this comment, we have revised the Discussion to clarify that this finding reflects a plateau in symptom resolution during the observation window and does not preclude the possibility of further improvement beyond the follow-up period.

   (Discussion) Page17, Line278-280

   Regarding duration, among adults, approximately 20% of pre-Omicron infections and about 10% of Omicron infections still had persistent symptoms two years after infection. In children, prevalence was substantially lower, with around 4% for pre-Omicron and 2% for Omicron infections. Symptoms persisting for more than two years rarely resolved thereafter. **This finding**

reflects the observed plateau in symptom resolution within the available follow-up period and does not preclude the possibility of further improvement beyond the observation window.

3. *Variant Attribution: Epidemic periods were defined using regional surveillance data rather than individual-level viral sequencing. While this approach is appropriate, some parts of the Discussion imply variant-specific biological effects. I suggest framing the findings more consistently as period-based associations and clearly presenting variant-related explanations as hypotheses rather than causal conclusions.*

   Response:
   We thank the reviewer for this constructive comment. In response, we revised the Discussion to more consistently frame our findings as period-based associations rather than causal effects. Specifically, the wording in the Delta-related paragraph was modified to clarify that variant-related biological explanations are presented as hypotheses rather than causal conclusions.

   (Discussion) Page 18, Lines 301–302
   The Delta variant carries the P681R mutation in the spike protein and exhibits enhanced cell–cell fusion capacity, both of which have been shown in epidemiological studies and animal models to contribute to increased disease severity (18). **Our findings are consistent with the potential clinical implications of these virological features.**

4. *Vaccination as an Unmeasured Factor: Vaccination status is not included in the current analysis, despite major differences in vaccine coverage across epidemic periods and age groups. The absence of vaccination data remains an important limitation in interpreting differences between Delta and Omicron periods. This should be more explicitly acknowledged, particularly when discussing faster symptom resolution during Omicron waves.*

   Response:
   We thank the reviewer for this important comment. We agree that vaccination may influence the risk of post-COVID-19 symptoms and acknowledge its relevance when interpreting differences across epidemic periods.
   In the revised manuscript, we have clarified this issue in the Discussion. Although vaccination status was not included in the present analyses, our previous analysis using the same cohort did not find a significant association between vaccination history and post-COVID-19 symptoms persisting beyond three months. We therefore noted that, while other studies have reported a protective association between vaccination and long COVID in different settings, such an association was not observed in our cohort.

   (Discussion, Limitations) Page 19-20, Lines344-351
   Fifth, the effects of vaccination were not directly evaluated in the present study. In our previous analysis using the same cohort (12), vaccination history was not significantly associated with post-COVID-19 symptoms persisting for more than three months after infection in multivariable models. **One possible explanation is that the study population included relatively few severe acute COVID-19 cases, which may have limited the ability to detect a protective effect of vaccination on long-term outcomes. Although other studies have reported an association between vaccination and a reduced risk of long COVID in different settings (5), such an**

association was not observed in our cohort.

Minor Comments:

1. *A brief justification for defining the outcome as "any symptom," despite wide variation in severity and clinical impact, would be helpful.*

   Response:
   We thank the reviewer for this helpful suggestion. We have clarified the rationale for defining the outcome as "any symptom" in the Method section, noting that this definition was used to capture the overall burden of persistent post-COVID-19 symptoms across epidemic periods and age groups.

   (Methods) Page 8, Lines146-147
   Outcome
   The primary outcome of this study was the duration of post-COVID-19 symptoms. For adults and children separately and across epidemic periods, we evaluated (1) the proportion of individuals reporting any level of severity, **defined as the presence of at least one post-COVID-19 symptom, to capture the overall burden of persistent symptoms,** and (2) the proportion reporting symptoms that interfered with daily life.

2. *The self-reported nature of "interference with daily life" may be interpreted differently across age groups; a short acknowledgment of this limitation would strengthen the methods.*

   Response:
   We thank the reviewer for this helpful comment. We have added a brief acknowledgment in the Discussion section noting that interference with daily life was self-reported and that its interpretation may have differed across age groups, particularly for pediatric participants assessed via proxy responses.

   (Discussion, Limitations) Page 20, Lines357-361
   Second, recall bias is possible, since the duration of symptoms was self-reported. To mitigate this, participants were asked to report duration in months, and interval-censored survival analysis using the Turnbull method was applied to enhance reliability. **In addition, interference with daily life was also self-reported, and its interpretation may have differed across age groups, particularly for pediatric participants assessed via proxy responses.**

3. *Terminology (post-COVID-19 symptoms, post-COVID-19 condition, long COVID) could be used more consistently throughout the manuscript.*

   Response:
   We thank the reviewer for this helpful comment. We have reviewed the entire manuscript, including the Introduction, to improve consistency in terminology. Throughout the manuscript, we now primarily use the term "post-COVID-19 symptoms," while the terms "long COVID" and "post-COVID-19 condition" are reserved for references to established definitions or prior literature. This clarification has been added to the Introduction.

   (Introduction) Page 5, Lines78-80

In this manuscript, we primarily use the term "post-COVID-19 symptoms" to describe persistent symptoms following SARS-CoV-2 infection, while the terms "long COVID" and "post-COVID-19 condition" are used when referring to established definitions or prior literature.
