# Supplementary material for: Differences in the long-term course of post-COVID-19 symptoms in adults and children across epidemic periods: A retrospective cohort study in Japan, 2020–2024
Source: PLoS One. 2026 May 8;21(5):e0348954. doi: 10.1371/journal.pone.0348954 (PMC13155679; doi:10.1371/journal.pone.0348954)
Supplement: Attachment — Submitted filename: Response to Reviewers_260408.pdf [file pone.0348954.s007.pdf]

Response to Reviewers

We sincerely thank the Academic Editor and the reviewers for their careful evaluation of the revised manuscript and for the constructive comments provided in this second round of review. We appreciate their continued engagement with our work, which has helped us to further strengthen the manuscript.

We have carefully considered all remaining comments and have revised the manuscript accordingly. Below, we provide a point-by-point response to each comment. All changes made to the manuscript are indicated in the revised version with tracked changes.

Reviewer #1

I would like to thank the authors for their detailed responses and the effort put into the revision. They have addressed all of my previous concerns satisfactorily. The manuscript is now much stronger and provides a valuable contribution to the field.

Response:

We sincerely thank Reviewer #1 for the thorough and constructive review of our manuscript. We are grateful for the positive assessment and encouraging comments. The reviewer's thoughtful feedback in the previous round of review contributed greatly to strengthening the manuscript, and we deeply appreciate the time and effort devoted to this evaluation.

Reviewer #5

In the current work, the authors present a retrospective analysis of the persistence of COVID-19 associated symptoms (long COVID) in patients diagnosed with COVID-19 during different time periods which were dominated by different SARS-CoV-2 variants. The study provides a long term evaluation of the COVID-19 associated symptoms with comparison across the old and newer variants and also adult and children study groups and thus provides an important dataset from a healthcare and immunological standpoint. A few suggestions/comments regarding the study are as follows:

1. *The study assigns different strains of SARS-CoV-2 to different years in which the patients were infected. While this is a largely accepted practice, a lack of sequencing information for the infecting viral strain still is a caveat while interpreting the study results.*

   Response:
   We thank the reviewer for this comment. We agree that a lack of sequencing information for the infecting viral strain is a caveat when interpreting the results, as the reviewer noted. To address this limitation, epidemic periods were classified based on publicly available variant surveillance data from Hiroshima Prefecture, which tracked the predominant circulating variants at the population level—a largely accepted practice in epidemiological studies of long COVID. In response to the reviewer's comment, we have revised the Limitations section to explicitly acknowledge this caveat, while also noting that any resulting misclassification would likely bias estimates toward the null, making overestimation of variant-specific differences unlikely.

   Original text
   (Discussion, Limitations) Page 19, Line 332–334:
   Third, genomic sequencing was not performed to identify the infecting variants, raising the possibility of some misclassification. Such misclassification would likely bias the results toward the null; therefore, the significant differences observed suggest that overestimation is unlikely.

   Revised text
   (Discussion, Limitations) Page 19, Line 335-342:
   Third, genomic sequencing was not performed on individual patient samples to identify the infecting variants. To address this limitation, epidemic periods were classified based on publicly available variant surveillance data from Hiroshima Prefecture, which tracked the predominant circulating variants at the population level—a largely accepted practice in epidemiological studies of long COVID. Nevertheless, a lack of sequencing information for the infecting viral strain remains a caveat when interpreting the results. Such misclassification would likely bias estimates toward the null; therefore, the significant differences observed suggest that overestimation is unlikely.

2. *In each of the category of patients, is there any information on the presence of vaccination and recovery from long COVID symptoms.*

   Response:

We thank the reviewer for this comment. Regarding vaccination history, data on vaccination status are available in our dataset. However, the association between vaccination history and post-COVID-19 symptoms was already examined and reported in our previous publication using the same cohort (reference 12). In that analysis, vaccination history was not significantly associated with post-COVID-19 symptoms persisting beyond three months in multivariable models. We wish to emphasize that this finding does not imply that vaccination has no effect on long COVID in general, but rather may reflect characteristics of our cohort, such as the relatively small number of severe acute COVID-19 cases. Given that the association between vaccination and long-term symptom outcomes had already been addressed in our prior work, and that the primary objective of the present study was to compare symptom trajectories across epidemic periods and between adults and children, a further detailed evaluation of vaccination effects was considered outside the scope of this study. We believe the Limitations section (Limitation 5) adequately addresses this point, but in response to the reviewer's comment, we have revised this section to more explicitly convey this rationale.

Original text
(Discussion, Limitations) Page 19-20, Line 340–347:
Fifth, the effects of vaccination were not directly evaluated in the present study. In our previous analysis using the same cohort (12), vaccination history was not significantly associated with post-COVID-19 symptoms persisting for more than three months after infection in multivariable models. One possible explanation is that the study population included relatively few severe acute COVID-19 cases, which may have limited the ability to detect a protective effect of vaccination on long-term outcomes. Although other studies have reported an association between vaccination and a reduced risk of long COVID in different settings (5), such an association was not observed in our cohort.

Revised text
(Discussion, Limitations) Page 20, Line 348-357:
Fifth, data on vaccination history were available in our dataset; however, the association between vaccination and post-COVID-19 symptoms was already examined in our previous analysis using the same cohort (12), in which vaccination history was not significantly associated with symptoms persisting beyond three months in multivariable models. This finding does not imply that vaccination has no effect on long COVID in general, but rather may reflect characteristics of our cohort, such as the relatively small number of severe acute COVID-19 cases. Given that this association had already been addressed in our prior work, a further detailed evaluation of vaccination effects stratified by epidemic period was considered outside the scope of the present study, which primarily aimed to compare symptom trajectories across epidemic periods and between adults and children.

3. *In the patients which require critical care, is there any co-relation between treatment strategies used and the better outcome in terms of long-term symptom alleviation.*

Response:
We thank the reviewer for raising this clinically important question. In the present study, the number of severe and critical cases was extremely limited (critical cases: n=3), precluding

any meaningful analysis of the relationship between treatment strategies and long-term outcomes in this subgroup. In response to the reviewer's comment, we have expanded the relevant Limitations section to explicitly acknowledge this issue and to identify it as an important direction for future research.

Original text

(Discussion, Limitations) Page 20, Line 362–364:

Eighth, the number of severe cases was limited, and thus disease courses specific to severe cases could not be fully evaluated. This issue should be addressed in other cohorts.

Revised text

(Discussion, Limitations) Page 21, Line 372-376:

Eighth, the number of severe and critical cases was limited (critical cases: n=3), and thus disease courses specific to severe cases could not be fully evaluated. Examining the relationship between acute treatment strategies and long-term symptom alleviation in critically ill patients is an important research question that should be addressed in future studies incorporating data from intensive care settings.

4. *In case of children as the reporting is done by the parents/supervising adults, the presence of symptoms which interfere with daily life might not be very accurate.*

Response:

We thank the reviewer for this important methodological point. We fully agree that proxy-reported outcomes in children may not accurately capture the presence or severity of symptoms that interfere with daily life, particularly for subjective symptoms—such as fatigue, difficulty concentrating, and sleep disturbances—that are not readily observable by caregivers. In response to the reviewer's comment, we have expanded the relevant Limitations section to more explicitly discuss the direction and potential impact of this bias.

Original text

(Discussion, Limitations) Page 19, Line 330–331:

In addition, interference with daily life was also self-reported, and its interpretation may have differed across age groups, particularly for pediatric participants assessed via proxy responses.

Revised text

(Discussion, Limitations) Page 19, Line 332-335:

In addition, interference with daily life was also self-reported, and its interpretation may have differed across age groups, particularly for pediatric participants assessed via proxy responses. Proxy-reported outcomes may underestimate the true prevalence of subjective symptoms—such as fatigue, difficulty concentrating, and sleep disturbances—that are not readily observable by caregivers. This limitation should be considered when interpreting the lower symptom prevalence and impact observed in children compared with adults.
