# Supplementary material for: Differences in the long-term course of post-COVID-19 symptoms in adults and children across epidemic periods: A retrospective cohort study in Japan, 2020–2024
Source: PLoS One. 2026 May 8;21(5):e0348954. doi: 10.1371/journal.pone.0348954 (PMC13155679; doi:10.1371/journal.pone.0348954)
Supplement: Attachment — Submitted filename: Review work.docx [file pone.0348954.s004.docx]

This study with the title "Differences in the Long-term Course of Post-COVID-19 Symptoms in Adults and Children across Epidemic Periods: A Retrospective Cohort Study in Japan, 2020–2024" gives an insight about the long-term effects of Covid 19 and its co-relation with patient age. But the study seems to have a few unanswered questions. The authors need to discuss some of these questions: -

1. The authors discussed the different strains of SARS Covid-19 and their long-term symptoms in different patient groups. Do these patients get more than one strain or are they affected by multiple waves of the infection at different time points?
2. The study is based on a survey that includes children who can have different experiences than the adult involved. How did the authors provide more information and support to the children for specific responses.
3. In figure 2, it is interesting to find old versions of viruses showing more severity in symptoms than the later mutants arise. Is it consistent with time and people in each survey.
4. I did not find any information about the co-infection or any other illness that happened to the responders during the survey duration.
