# Supplementary material for: Differences in the long-term course of post-COVID-19 symptoms in adults and children across epidemic periods: A retrospective cohort study in Japan, 2020–2024
Source: PLoS One. 2026 May 8;21(5):e0348954. doi: 10.1371/journal.pone.0348954 (PMC13155679; doi:10.1371/journal.pone.0348954)
Supplement: Attachment — Submitted filename: Review.docx [file pone.0348954.s006.docx]

In the current work, the authors present a retrospective analysis of the persistence of COVID-19 associated symptoms (long COVID) in patients diagnosed with COVID-19 during different time periods which were dominated by different SARS-CoV-2 variants. The study provides a long term evaluation of the COVID-19 associated symptoms with comparison across the old and newer variants and also adult and children study groups and thus provides an important dataset from a healthcare and immunological standpoint. A few suggestions/comments regarding the study are as follows:

1. The study assigns different strains of SARS-CoV-2 to different years in which the patients were infected. While this is a largely accepted practice, a lack of sequencing information for the infecting viral strain still is a caveat while interpreting the study results.

2. In each of the category of patients, is there any information on the presence of vaccination and recovery from long COVID symptoms.

3. In the patients which require critical care, is there any co-relation between treatment strategies used and the better outcome in terms of long-term symptom alleviation.

4. In case of children as the reporting is done by the parents/supervising adults, the presence of symptoms which interfere with daily life might not be very accurate.
